# Effect of Glycero-(9,10-trioxolane)-trialeate on the Physicochemical Properties of Non-Woven Polylactic Acid Fiber Materials

**DOI:** 10.3390/polym13152517

**Published:** 2021-07-30

**Authors:** Anatoliy Olkhov, Olga Alexeeva, Marina Konstantinova, Vyacheslav Podmasterev, Polina Tyubaeva, Anna Borunova, Valentina Siracusa, Alex L. Iordanskii

**Affiliations:** 1 N.M. Emanuel Institute of Biochemical Physics, Russian Academy of Sciences, Kosygin St. 4, 119991 Moscow, Russia; aolkhov72@yandex.ru (A.O.); kisisinova@yandex.ru (M.K.); vpodmasterev@yandex.ru (V.P.); polina-tyubaeva@yandex.ru (P.T.); 2 Department of Chemistry and Physics, Plekhanov Russian University of Economics, Stremyanny Ln 36, 117997 Moscow, Russia; 3 N.N. Semenov Federal Research Center for Chemical Physics, Russian Academy of Sciences, Kosygin St. 4, 119334 Moscow, Russia; aborunova@mail.ru; 4 Department of Chemical Science (DSC), University of Catania, Viale A. Doria 6, 95125 Catania, Italy

**Keywords:** polylactic acid, electrospinning, ozonide, trioleate, biocompatibility

## Abstract

Biocompatible glycero (9,10-trioxolane) trioleate (ozonide of oleic acid triglyceride, OTOA) was incorporated into polylactic acid (PLA) fibers by electrospinning and nonwoven PLA mats with 1%, 3% and 5% OTOA content. The morphological, mechanical, thermal and water sorption properties of electrospun PLA mats after the addition of OTOA were studied. A morphological analysis showed that the addition of OTOA increased the average fiber diameter and induced the formation of pores on the fiber surface, leading to an increase in the specific surface area for OTOA-modified PLA fibrous mats. PLA fiber mats with 3% OTOA content were characterized by a highly porous surface morphology, an increased specific surface area and high-water sorption. Differential scanning calorimetry (DSC) was used to analyze the thermal properties of the fibrous PLA mats. The glass transition temperatures of the fibers from the PLA–OTOA composites decreased as the OTOA content increased, which was attributed to the plasticizing effect of OTOA. DSC results showed that OTOA aided the PLA amorphization process, thus reducing the crystallinity of the obtained nonwoven PLA–OTOA materials. An analysis of the mechanical properties showed that the tensile strength of electrospun PLA mats was improved by the addition of OTOA. Additionally, fibrous PLA mats with 3% OTOA content showed increased elasticity compared to the pristine PLA material. The obtained porous PLA electrospun fibers with the optimal 3% OTOA content have the potential for various biomedical applications such as drug delivery and in tissue engineering.

## 1. Introduction

Eco-friendly, biodegradable polymer materials produced in the form of ultrathin fibers are of growing importance due to their exceptional properties such as high surface/volume ratio, biocompatibility, non-toxicity and potential for application in drug delivery systems, tissue engineering and regenerative medicine. The successful application of polymer materials in biomedical applications should be based on a number of properties such as adequate mechanical strength, improved elasticity, the lack of ability to elicit allergic reactions, good biodegradability, etc. [1,2,3,4].

One of the promising biopolymers widely used in various applications such as biomedicine [5], packaging [6] and environmental protection is polylactic acid (PLA), which is produced through the enzymatic fermentation of renewable natural products such as corn, potato, sugar, etc. PLA could be extruded into films and pellets and formed via electrospinning into ultrafine fiber mats.

Electrospinning is a facile and versatile technique for obtaining the micro- and nano-sized fibers. The electrospinning of functional polymer nanofibers has attracted a lot of attention recently due to the simplicity of the process and the improved properties associated with the morphology of the resulting fibers [7,8]. Fibrous nanoscale materials formed during electrospinning have a high porosity and a large specific surface area, which allows them to be used as highly efficient filters and absorbents [9]. Additionally, electrospun polymer materials could be easily modified with different biologically active compounds [10], biomolecules [11] and carbon nanomaterials such as carbon nano-onions [12,13] to provide the surface functionalization of polymeric electrospun scaffolds for drug delivery, regenerative medicine and tissue engineering.

The fabrication of electrospun ultra-thin nanofibers and fiber membranes based on PLA and blends with other biopolyesters has been widely investigated recently [14,15,16,17]. Fibrous electrospun materials produced from eco-friendly polymers, such as PLA, have all the necessary properties of biocompatibility, biodegradability and non-toxicity for use in various biomedical, packaging and environmental applications [18,19,20,21]. However, PLA application is limited by its mechanical and physicochemical properties, including brittleness, poor elasticity, low water sorption due to hydrophobicity and relatively low thermostability. One of the approaches to resolve these issues is the modification of PLA materials with functional additives which improve the initial properties of PLA and provide additional functionality for various biomedical applications [19,22]. Introduced additives should have a good compatibility with the polymer and should be safe for use in biomedical applications. Recent papers have reported on the production and characteristics of electrospun fibers based on PLA with different polymer additives [23], as well as PLA blends with poly(3-hydroxybutyrate) (PHB) and other polymers [24,25]. These reports show that polymer additives could improve the uniformity in the distribution of PLA fibers and increase the porosity and specific surface area of nonwoven electrospun PLA-based materials.

It should be noted that the combination of PLA with other polymers in the binary blends often requires the use of plasticizers such as limonene [26], polyethylene glycol (PEG) [27] and others. Due to strict requirements in food packaging and biomedical applications, novel biocompatible and non-toxic plasticizing agents should be provided in order to improve the initial properties of the electrospun PLA-based materials. The plasticizing properties of vegetable and mineral oils have been used recently to improve the properties of polymer materials [28,29]. Moreover, ozonated vegetable oils are even more promising in this respect since they could combine plasticizing properties together with apparent antimicrobial activity [30]. The methods for producing ozonized vegetable oils are well known and have been described in detail [30,31]. Ozone is a strong oxidizing agent that can react with unsaturated triacylglycerols found in vegetable oils. The ozonation of olefins is usually viewed in terms of the mechanism postulated by Criegee [32]. This mechanism describes the reaction of ozone with an unsaturated bond, with the formation of the initial unstable primary ozonide molecule (R-C-O_3_-C-R′). This primary ozonide readily decomposes to form zwitterion and carbonyl moiety. These fragments can then combine to form the trioxolane compound. The most interesting of these products is OTOA, the ozonide of oleic acid triglyceride, which is the major triglyceride (>45%) in olive oil. OTOA, which is non-toxic, biocompatible and a biodegradable material [33,34], could be a promising additive for introduction into the PLA matrix via electrospinning.

In this study, electrospinning was used to fabricate nonwoven PLA mats with different OTOA contents (1%, 3% and 5% *w*/*w*). Morphological, mechanical, thermal and water sorption properties of electrospun PLA mats were tested using optical and scanning electron microscopy (SEM), differential scanning calorimetry (DSC) and FTIR spectroscopy to provide deeper insights into the specific PLA–OTOA interactions and the effect of the OTOA content on the physicochemical properties of nonwoven PLA materials. The study demonstrates that electrospun PLA fibers with added OTOA have a highly porous surface morphology, an increased specific surface area, a high water sorption and improved mechanical properties over pristine electrospun PLA. Therefore, they have the potential for many applications such as tissue engineering, drug delivery, filtration, etc.

## 2. Materials and Methods

**Figure 1:**
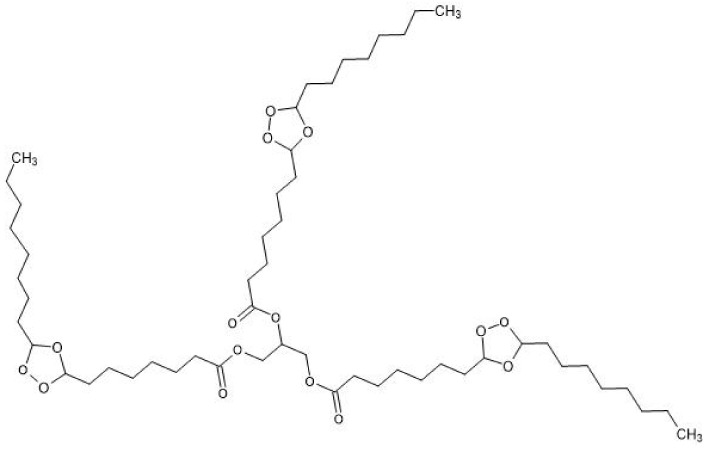
Chemical structure of glycero-(9,10-trioxolane)-trioleate (ozonide of oleic acid triglyceride, OTOA).

PLA was purchased by NatureWorks^®^ Ingeo™ 3801X Injection Grade from Shenzhen Bright China Inc. (Shenzhen, China) with a viscosity average molecular weight of 1.9 × 10^5^ g/mol. Dry clean chloroform ≥99.5%, used for the preparation of molding solutions, Sigma-Aldrich Inc. (Saint Louis, MO, USA); Glycero-(9,10-trioxolane)-trialeate (ozonide of oleic acid triglyceride (OTOA)) was obtained by Medozon (Moscow, Russia), reported in Figure 1. All reagents were used as received.

The chemical structure and physicochemical properties of OTOA has been previously studied in recent papers [30,35] using NMR. ^1^H and ^13^C NMR spectra showed that all the C=C bonds in triacylglycerols were converted into ozonide. The spectra did not show any other structural changes or the absence of the products of ozonide decomposition, such as aldehydes and carbon acids. Moreover, a good stability at room temperature and antimicrobial activity have been shown for OTOA [30].

**Figure 2:**
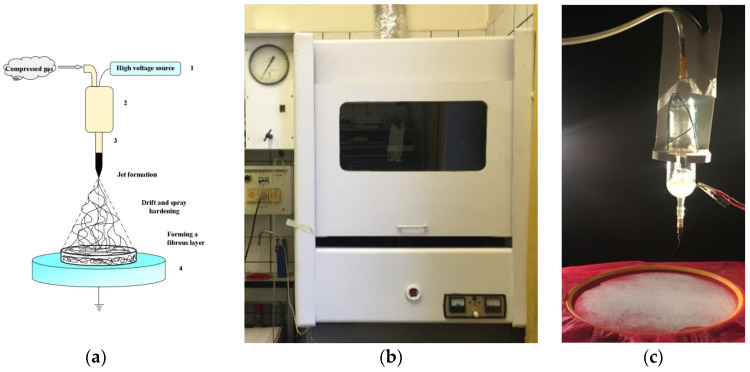
Schematic illustration showing the electrospinning process (**a**), general view of the experimental setup (**b**) and the process of obtaining ultrafibrous material based on PLA (**c**).

Using the electrospinning method (Figure 2a), nonwoven ultrathin fiber materials were obtained from a 10% PLA solution in chloroform, with the addition of OTOA at concentrations of 1, 3 and 5 wt.%. In order to obtain ultrathin fibers by the electrospinning method, an original single-capillary laboratory setup “EPV-1” (Federal Research Center of Chemical Physics RAS, Moscow, Russia) was used, shown in Figure 2b,c, with the following technological parameters: a capillary diameter of 0.1 mm, a voltage of 15 kV, a distance between electrodes of 18 cm and a solution conductivity of 10 μS/cm. The amount of the residual chloroform in the PLA fibers was quantified by drying the PLA mats in a vacuum oven at 25 ℃ up to the constant weight. Negligible residual solvent content was obtained for all studied PLA samples.

The analysis of the nonwoven material by FTIR spectroscopy was carried out on an FTIR PERKIN-ELMER Spectrum Two setup (Waltham, MA, USA) with a modified DRIFT attachment (Diffuse Reflectance Infrared Fourier Transform Spectroscopy), allowing the measurements of solid samples [36,37]. A small 13 mm diameter stainless steel table acting as a mirror was used and the infrared ray penetrated the sample placed directly on the table surface. The position of the sample holder was optimized prior to measurement, using a clean metal roller. FTIR spectra were recorded in the 4000–400 cm^−1^ range, with a resolution of 4 cm^−1^ and an average of 16 successive scans.

The thermophysical properties of the fibrous materials were obtained using a NETZSCH DSC 204F1 Phoenix differential scanning calorimeter (Selb, Germany) in an inert atmosphere at a 100 mL/min argon flow rate. Fiber mat samples (approximately 5 mg) were placed in aluminum sample pans and heated in the temperature range from 20 °C to 200 °C at a 10 °C/min heating rate. The instrument was calibrated against indium, tin and lead. After the first heating cycle, samples were kept at 200 °C for 5 min, cooled to room temperature and then reheated to 200 °C at 10 °C/min to record the second DSC curve. All samples were tested in triplicate.

The morphology and the diameter of the fibers were analyzed using a Hitachi SU8000 scanning electron microscope (SEM) (Hitachi High Technologies, Tokyo, Japan) at an accelerating voltage of 1 kV. Samples were mounted on a 25 mm aluminum specimen stub and fixed by graphite adhesive tape. A metal coating with a thin film (10 nm) of platinum was performed using magnetron sputtering. The diameters of the fibers were measured directly from the SEM images using ImageJ software, with the average fiber diameter and size distribution calculated from the measurements of at least 200 fibers.

The specific surface area (S) of nonwoven fabrics without and with 1%, 3% and 5% OTOA, respectively, was analyzed by the Brunauer–Emmett–Teller (BET) multipoint method using nitrogen adsorption/desorption isotherm measurements at −196 °C, by the nitrogen adsorption instrument ASAP 2010 (Micromeritics Instruments, Norcross, GA, USA).

To determine water sorption [38], nonwoven samples were cut into uniform strips of 4 × 4 cm and conditioned under standard atmospheric conditions (44 °C ± 2% RH and 20 °C ± 2 °C) for 24 h. After conditioning, the weight (m_1_) was measured. Then, samples were immersed in distilled water for 24 h at a temperature of 20 °C ± 2 ° C to ensure uniform water saturation. Thereafter, the wet samples were hung in open air at 20 °C ± 2 °C for 30 min to remove water excess on the surface of the samples. Then, the weight (m_2_) of the wet samples was measured. The moisture content of the samples was calculated by the formula:

Q = (m_2_ − m_1_)/m_1_ × 100% (1)

with m_1_ and m_2_ being the sample weight before and after immersion in liquid.

The degree of hydrophilicity of the samples was determined by the water contact angle measurements in its semi-angle modification [39] for a drop of water placed on the material surface. The contact angle (or wetting angle) is the angle formed by the tangent planes to the interfacial surfaces that limit the wetting liquid and the apex of the angle lies on the separation line of the three phases. The contact angle θ or cosθ is a characteristic of the hydrophilicity (hydrophobicity) of the surface. Since in our case the droplet size was small, it can be represented as a part of a sphere and a droplet profile in two dimensions, i.e., in the form of a circle.

**Figure 3:**
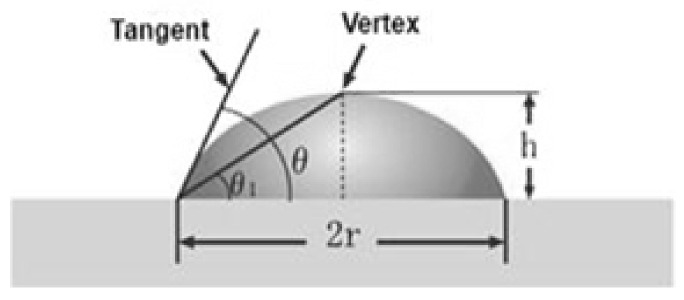
Scheme for the measurement of the average contact angle: h is the height and r is half the width of the baseline.

In general, *θ*_1_ = *tan*^−1^ *h*/*r*, but in the case of simple geometry, the contact angle can be expressed as follows *θ* = 2*θ*_1_ (see Figure 3). By calculating *h* and *r* using image analysis, the contact angle θ can be calculated. The fibers of the original PLA and PLA with the addition of OTOA (1%, 3%, 5%) were pulled out of the material and distilled water was sprayed on them. The droplet size measurements were carried out using an OLIMPUS CX21 optical microscope (Tokyo, Japan) equipped with a digital camera. The results were processed using the MICAM 3.02 software.

The surface density of the samples ρ_s_ (g/m^2^) was calculated by the equation:

ρ_s_ = m/L ∗ B (2)

where m is the mass of the sample, expressed in grams (g); L is the sample length, expressed in meters (m); and B is the sample width, expressed in meters (m).

The bulk density of the samples ρ_v_ (g/cm^3^) was calculated by the equation:

ρ_v_ = m/V (3)

where m is the mass of the sample, expressed in grams (g) and V is the volume of the sample, expressed in cubic centimeters (cm^3^).

The determination of the open volume of the capillaries for the nonwoven fabric V_C_ (cm^3^) and the proportion of the open volume of the capillaries to the total volume of the material W_C_ was carried out according to the following equation:

V_C_ = (m_2_ − m_1_)/ρ_l_ (4)

W_C_ = V_C_/V·100% (5)

where m_1_ is the mass of the dry sample; m_2_ is the mass of the sample after immersion in liquid, expressed in grams (g); ρ_l_ is the density of the liquid (distilled water); and V is the volume of the sample, expressed in cm^3^.

The mechanical characteristics of the samples were determined on an Instron-3365 tensile machine (High Wycombe, UK) under uniaxial stretching, at the rate of the upper traverse motion of 50 mm/min at room temperature. Strips of 7.5 cm × 1 cm size were used as samples for the mechanical tests. From the obtained diagrams, tensile strength and elongation at the break were calculated. All samples were tested in triplicate.

For the statistical analysis, a one-way ANOVA was applied. All the experiments were run in triplicate and the data were presented as mean value ± standard deviation at a significance level of *p* < 0.05.

## 3. Results

### 3.1. Morphology

**Figure 4:**
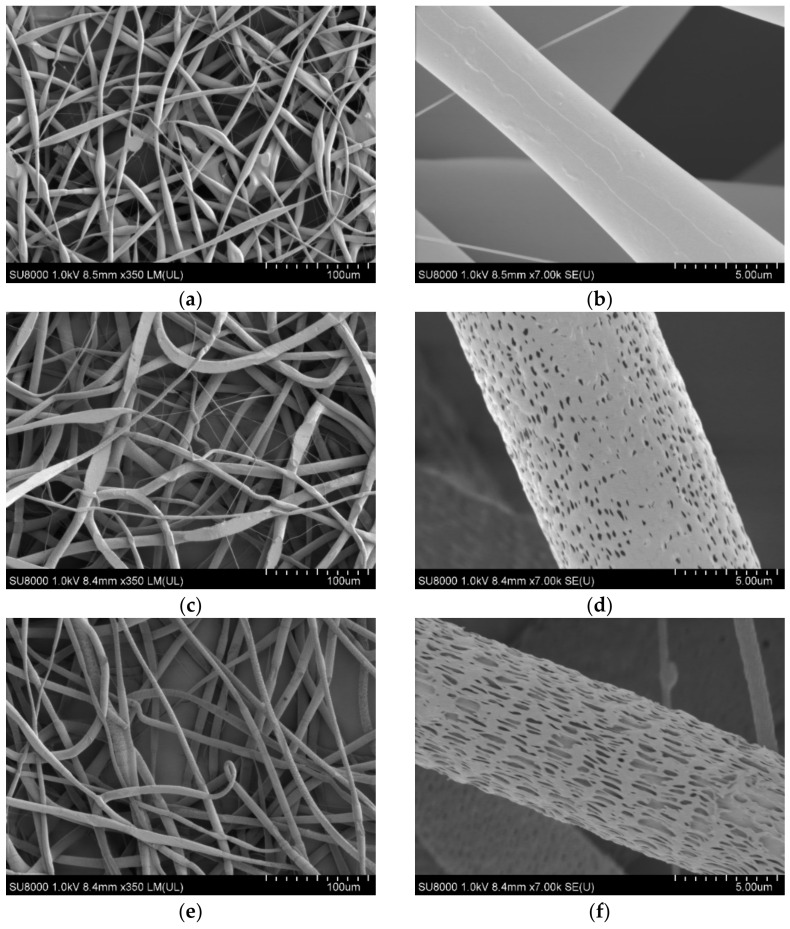

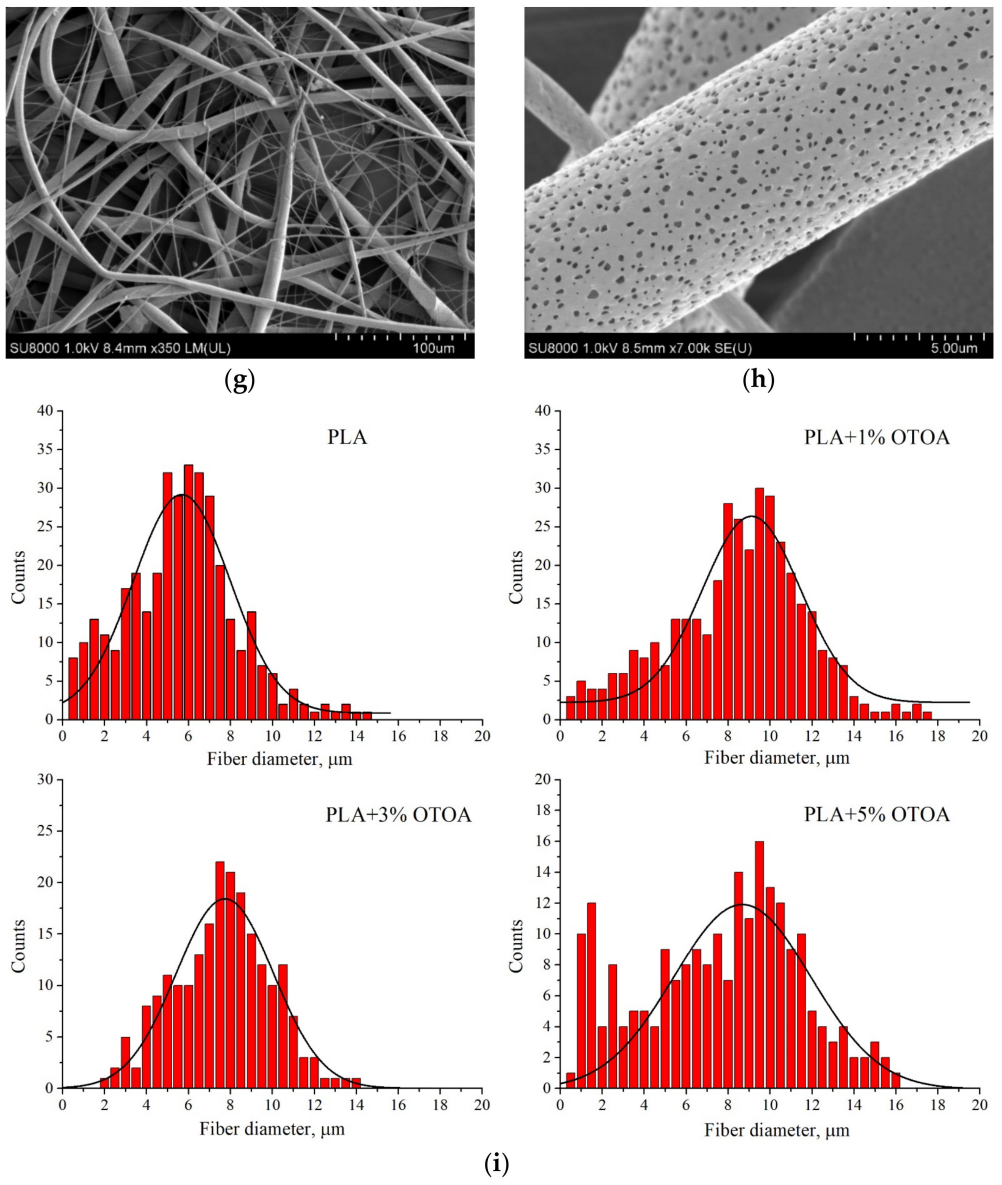
SEM images of nonwoven fiber PLA mats and magnified monofilaments: pristine PLA (**a**,**b**), PLA + 1% OTOA (**c**,**d**), PLA + 3% OTOA (**e**,**f**), PLA + 5% OTOA (**g**,**h**). Distributions of fiber diameters for studied PLA fibrous samples (**i**).

The morphological features of non-woven ultrathin fibrous materials (hereinafter-mats) are shown in Figure 4. The pristine PLA mat consisted of thin fibers with a diameter of 5–14 microns, characterized by noticeable sinuosity and the presence of small areas with local thickenings of 20–25 microns in the transverse and 100–150 microns in the longitudinal directions, as well as thinner sections with a diameter of 0.6–4 microns (Figure 4a). A detailed study of the surface of the pristine PLA fibers (Figure 4b) showed a smooth morphology and microrelief in the form of well orientated axially alternating bands. Apparently, the observed texture was formed on the fiber surface during its spinning, due to electrostatic and viscoelastic interactions realized on the fiber surface.

The results of the fiber diameter measurements for the nonwoven PLA fiber mats with different OTOA contents are presented in Table 1 and the fiber diameter distributions are shown in Figure 4i. As can be seen, the pristine PLA fiber mats showed an average fiber diameter of 5.7 ± 2.3 μm.

The effect of the addition of OTOA was manifested in the increase in the average fiber diameter from 5.7 μm to 8–9 μm, observed for all OTOA-containing samples. Additionally, ultra-thin micro- and submicro-sized fibers were detected, with diameters in the range of 0.6–2.0 microns. The greatest content was observed for the PLA fiber mat with 5% OTOA content, whereas the PLA fiber material with 3% OTOA showed the most uniform fiber size distribution (Figure 4i). An increase in the uniformity of distribution for electrospun PLA fibers upon the addition of the polymer was previously observed for PLA/chitosan composites [23].

The presence of two groups of fibers with a diameter in the micron range (4–16 μm) and ultra-thin fibers with a diameter of 0.6–2.0 μm could be explained by the effect of the partial splitting of the primary jet formed by the polymer solution. In the presence of a large organic compound, such as OTOA, the viscosity of the spinning solution increases, which facilitates the onset of the heterogeneity of the effluent solution and its decomposition into two fractions, thicker (micron) and thinner (submicron).

The surface morphology of PLA fibers containing OTOA differs from that of pristine PLA. The latter showed an absence of pores in the high magnification SEM image (Figure 4b). PLA samples with the addition of OTOA clearly show the presence of a nano-patterned surface, including mesopores distributed over the entire surface of the fiber (Figure 4d–h). The shape of the inlet of these pores was rather arbitrary and the dimensions lay in a narrow range of 0.2–1 μm. Apparently, the origin of the pores was primarily due to the desorption of the solvent from the bulk of the fiber and its subsequent rapid evaporation. The desorption of the solvent was determined by diffusion processes directly related to the viscosity of the polymer system in an inverse proportional relationship within the framework of the Stokes–Einstein model [40]. In the previous report, neat PLA fibers showed a highly porous morphology without any additives [25] which is in contrast to the results obtained in the present work. This could be attributed to the difference in the viscosity of the electrospinning solution, namely, pure chloroform (this work) and chloroform/DMF 9/1 *v*/*v* solution [25].

The closure of the fiber pores formed during the evaporation of the solvent occurs mainly by the mechanism of segmental motion, which was observed in PLA in the absence of OTOA [41]. With an increase in viscosity due to the addition of OTOA, the rate of closure/healing of the porous structure decreased and its morphology was fixed more effectively when the temperature of the polymer fiber was close to its glass transition temperature (T_g_). According to our previous studies that used the DSC method, T_g_ for PLA is near 65 °C [42] and, therefore, during electrospinning at room temperature, PLA passes through all physical states from viscous–fluid to glassy with a fixation of surface pores (see Figure 4d–h). Consequently, the difference in the surface morphology of PLA fibers with OTOA from the pristine PLA fibers is due to the presence of intermolecular interaction between the components of the spinning solution at the stage of fiber formation.

Recently, a novel Forcespinning^®^ (FS) technology was applied to produce micron and submicron polymeric fibers [43]. FS, which uses centrifugal forces to promote fiber formation, overcomes many intrinsic limitations of electrospinning, including the use of a high-voltage electric field (>10 kV), a requirement for the conductivity of the solution and a low fiber production rate. Although the FS technique is highly versatile and could provide high throughput polymer fiber production, the resulting morphology and the diameter of fibers depends on a large number of parameters, such as viscoelasticity and surface tension of the solution, nozzle geometry, rotation speed and polymer concentration [44]. On the other hand, it is known that the surface tension and the viscosity of the solution are the main parameters which codetermine the fiber morphology during electrospinning [25]. Other parameters (voltage, distance from the collector, needle diameter) influence the fiber morphology to a lesser degree. In recent papers, Forcespinning^®^ technology has been used to produce nano- and microsized PLA fibers and the optimal processing conditions were determined [45,46]. Thus, it was shown that both polymer concentration and rotation speed influence the PLA fiber morphology, with the average fiber diameter decreasing with an increase in rotation speed. Moreover, lower polymer concentrations and lower spinneret speeds favor a high degree of beading [45]. FS produces PLA nanofibers with diameters in the range of 200–600 nm, leading to a high surface-to-volume ratio. The conventional electrospinning process utilized in this work gave higher average PLA fiber diameters (above 1 μm), whereas a high specific surface area could be achieved through the porous fiber morphology observed upon OTOA addition.

Along with the morphological characteristics of PLA fibers obtained by SEM, an analysis of the fiber porosity parameters was carried out by the BET method, the water sorption measurements and calculations using Equations (2)–(5). The results are presented in Table 2. As follows from the table, when OTOA was added to the PLA fibers, the total porosity of the nonwoven material increased rapidly, which was manifested in the drastic increase in V_C_ and W_C_ values. The maximum porosity value was observed for the PLA mat with 3% OTOA content. The observed increase in the porosity of the PLA mats after the addition of OTOA is a consequence of two factors: (1) the presence of fibers of different diameters in the mat, which increase the interfibrillar volume due to the violation of the packing density; (2) the formation of the submicro- and nanoscale pores in the fibers due to the addition of OTOA. The subsequent increase in the OTOA content to 5% led to a significant decrease in the porosity of the PLA mats, which was shown by the decrease in V_C_, W_C_ and S values (Table 2).

**Figure 5:**
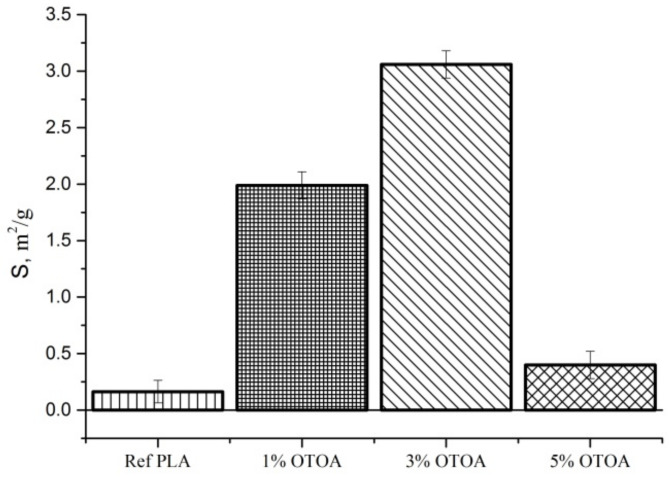
Specific surface area of nonwoven PLA mats: pristine PLA, PLA + 1% OTOA, PLA + 3% OTOA, PLA + 5% OTOA samples.

The effect of the porosity increase was confirmed by specific surface area measurements of the fibrous PLA mats, which were provided by the nitrogen adsorption method (BET method). As can be seen from Figure 5, the specific surface area of the fibrous material (S, m^2^/g) significantly increased upon the addition of OTOA, reaching the maximum value of 3.1 m^2^/g at 3% OTOA content. However, a further increase in OTOA content up to 5% led to a sharp decrease in the specific surface area. The observed significant drop in the S value for the PLA + 5% OTOA sample could be associated with the small size of the pores in the PLA fibers (Figure 4h) and the increase in the total volume of fibers could be due to the swelling and self-healing of the pores.

Based on the data shown in Figure 5 and Table 2, it could be concluded that the morphology of the developed PLA fibrous materials modified with OTOA has a complex hierarchical structure and includes a porous space between the fibers, the submicro- and micro-sized fibers and the pores in the fibers of submicrometer size. All this taken together indicates the formation of a heteroporous structure for PLA mats with added OTOA. As could be observed from Table 2, the introduction of OTOA affects the value of the open volume of the capillaries of the nonwoven fabric (V_C_) and the proportion of the open volume of the capillaries to the total volume of the material (W_C_), i.e., those characteristics that are largely responsible for the kinetic profile of controlled drug release [47].

The ultrathin fibers obtained in this work are mainly intended for use in various medical applications in the form of matrices and mats for the controlled release of biologically active substances [48], scaffolds in tissue engineering [49,50], artificial bioresorbable implants [51] and so on. Since all these applications require the direct contact of fibrous materials with various water-containing physiological media, the next stage of the work involved studying the hydrophilicity of the PLA–OTOA fibrous matrices.

**Figure 6:**
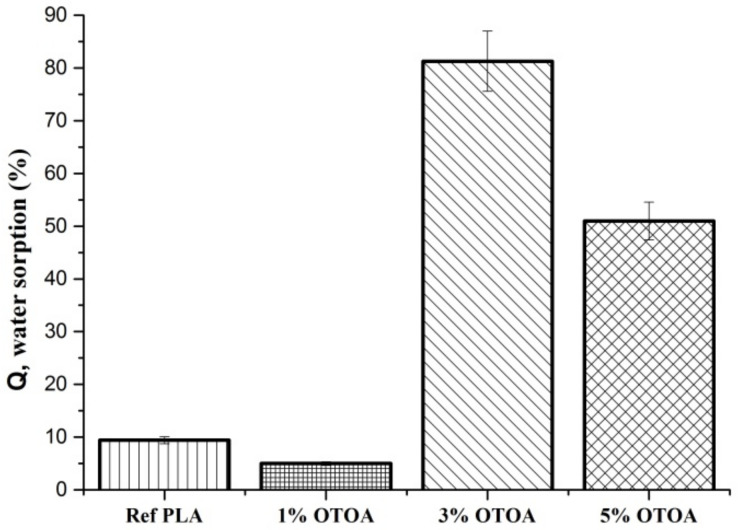
Sorption capacity (Q) of nonwoven PLA mats: pristine PLA, PLA + 1% OTOA, PLA + 3% OTOA, PLA + 5% OTOA samples.

As shown in Figure 6, the changes in the amount of absorbed water due to the OTOA modification of the PLA fibers depend on the OTOA concentration. Thus, the pristine PLA mat and the PLA + 1% OTOA samples were characterized by a lower water sorption capacity compared to PLA mats containing 3% and 5% OTOA. In the first approximation, the significant increase in water sorption capacity observed for PLA mats with 3% and 5% OTOA content is in satisfactory correlation with the results presented above for the porosity of nonwoven fibrous materials (Figure 4 and Table 2). With an increase in the total porosity of the fiber, its water sorption capacity grew symbatically. However, for the PLA fibrous material with 1% OTOA content, the correlation between porosity and water sorption was violated, leading to even lower Q values as compared to the pristine PLA mats (Figure 5 and Figure 6). Thus, a significant increase in porosity did not lead to an increase in sorption capacity for the PLA + 1% OTOA sample. It could be assumed that the observed effect could be ascribed to the morphology of the nonwoven PLA mats. As can be seen from Figure 4, the pristine PLA and the PLA +1% OTOA samples showed a dense packing of individual fibrils and a low interfibrillar volume. This leads to poor water diffusion into the porous medium, which prevents water from filling the pores in the individual fibers and leads to poor water sorption. In addition, it is known from the previous publications [52,53,54,55] that PLA exhibits moderate hydrophobic properties due to the low affinity of water for complex polyester groups, which prevents water sorption. Due to the chemical structure of PLA, containing low-polar ester groups, this biopolyester has low water solubility (in the fractions of a percent range). Therefore, according to the Reitlinger classification, PLA belongs to the moderately hydrophobic polymers [55]. At the same time, the structure of the OTOA molecule (Figure 1) also consists mainly of hydrophobic fragments and includes, along with low-polar ester groups, specific ozonide cycles. Thus, the addition of low amounts of OTOA under the certain threshold (1% in our case) does not lead to significant changes in the water sorption capacity of PLA due to the dense morphology of the nonwoven PLA mats and the hydrophobic nature of OTOA.

**Figure 7:**
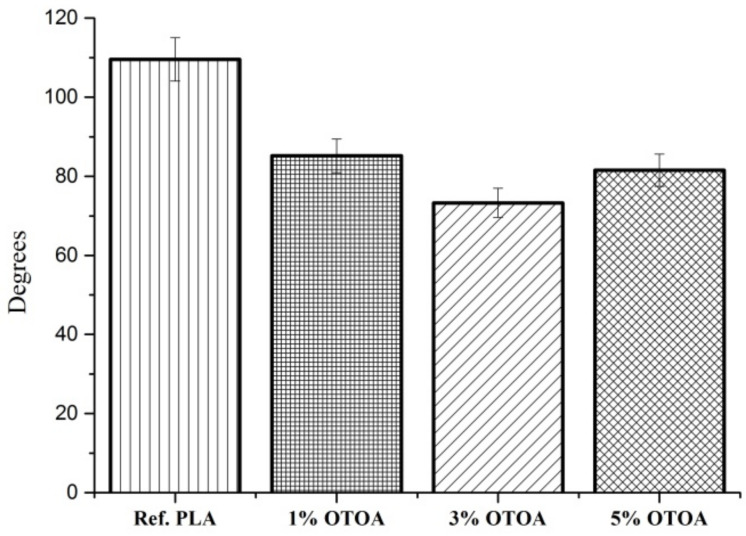
Water contact angle for nonwoven PLA materials: pristine PLA, PLA + 1% OTOA, PLA + 3% OTOA, PLA + 5% OTOA.

In order to determine the hydrophilic–hydrophobic properties of the fiber surface, the water contact angle was measured for the PLA fibrous mats with variable OTOA contents. Figure 7 demonstrates that in case of the reference PLA that did not contain OTOA, a poor wetting of the surface was observed, the droplet had an almost spherical shape, a weak interaction with the surface was observed and the contact angle was noticeably greater than 90°. With the introduction of OTOA, the contact angle decreased significantly and, considering the measurement error, remained practically unchanged at all OTOA concentrations. The results of the water contact angle measurements suggest that the introduction of the OTOA into the PLA fiber leads to a certain increase in the polarity of the PLA–OTOA fiber surface. The reason for this effect may be the distribution of long hydrophobic fragments of OTOA in the hydrophobic regions of the PLA fiber and the appearance of its oxygen-containing groups on the fiber surface exposed to water, i.e., additional hydrophilization of the PLA fiber due to ozonide addition.

### 3.2. FTIR Spectroscopy

**Figure 8:**
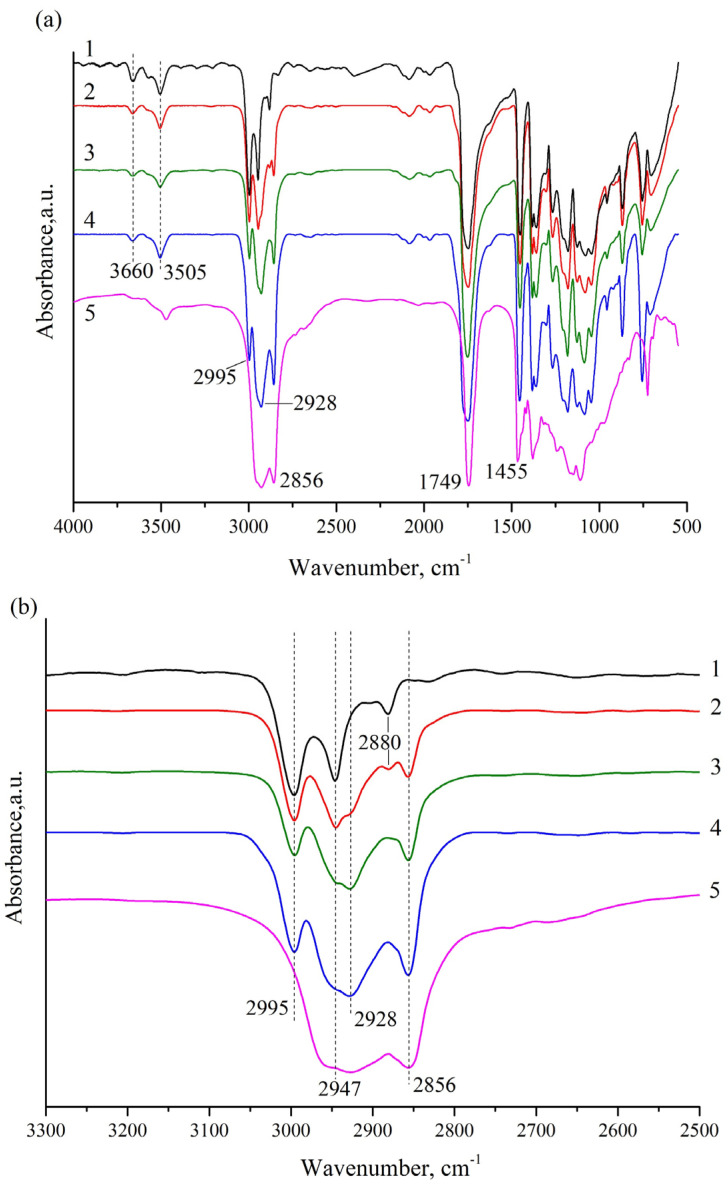
(**a**) FTIR spectra of the pristine PLA fiber mat (1), PLA + 1% OTOA (2), PLA + 3% OTOA (3), PLA + 5% OTOA (4), OTOA (5); (**b**) Close-up view of FTIR spectra in the vicinity of 3000 cm^−1^.

To provide more insight into the chemical interactions between PLA and OTOA, FTIR spectra of the nonwoven PLA materials were obtained (Figure 8a,b). The assignments of the main spectral bands in the FTIR spectra are presented in Table 3. The FTIR spectrum of the pristine PLA showed characteristic bands at 1455 cm^−1^ and 1749 cm^−1^ arising from the –CH_3_ bending vibration and the stretching vibration of the C=O groups. The absorption bands at 2947 and 2995 cm^−1^ are attributed to an asymmetrical stretching vibration of a –CH group. The peaks at 3505 and 3660 cm^−1^ could be attributed to the bending vibration of the terminal hydroxyl groups [56]. As can be seen in Figure 8a, the absorption bands of PLA and OTOA were practically in the same regions of the spectrum and were partially overlapped, which significantly complicates the analysis of the addition of small amounts of OTOA in the PLA fibers. However, as can be seen from Figure 8b, two additional bands at 2928 cm^−1^ and 2856 cm^−1^ appeared in the FTIR spectra for the PLA samples with added OTOA. These bands could be attributed to the symmetrical and asymmetrical stretching vibrations of the –CH_2_ group, with is abundant in OTOA (Figure 1). Since PLA does not contain –CH_2_ groups in the chemical structure, the appearance of these bands is clear evidence of OTOA incorporation into the PLA fibers. As can be seen from Figure 8b, the intensity of absorption band at 2856 cm^−1^ showed a good correlation with the OTOA content in the PLA fibers. Additionally, the 1745 cm^−1^ band in the pristine PLA shifted to higher wavenumbers (1751 cm^−1^) for the PLA samples with added OTOA (see Table 3). This indicates the weak interactions between the C=O groups of PLA and the polar ozonide groups in the OTOA molecule [56].

Additional information on the interactions between PLA and OTOA can be deduced from the analysis of the absorption bands at 3505 and 3660 cm^−1^. As can be seen from Figure 8a, the absorption peak at 3660 cm^−1^ showed a significant decrease after the addition of OTOA, whereas the 3505 cm^−1^ band became broader for the PLA–OTOA samples as compared to the pristine PLA. The obtained results provide evidence of the intermolecular interaction between PLA and OTOA, presumably between the terminal –OH groups in PLA and the polar ozonide groups in the OTOA molecule. Weak molecular interactions between PLA and different additives have been reported in previous works [56,57].

Based on the analysis of FTIR spectra, it can be assumed that weak interactions between the polar groups in PLA and OTOA can induce conformational changes in PLA chains associated with the reorientation of polar groups in PLA, thus facilitating the segmental mobility of PLA chains [42,56]. Thereby, low molecular weight OTOA could act as a plasticizer for the PLA polymer, affecting the thermal and mechanical properties of fibrous PLA materials.

### 3.3. Differential Scanning Calorimetry

**Figure 9:**
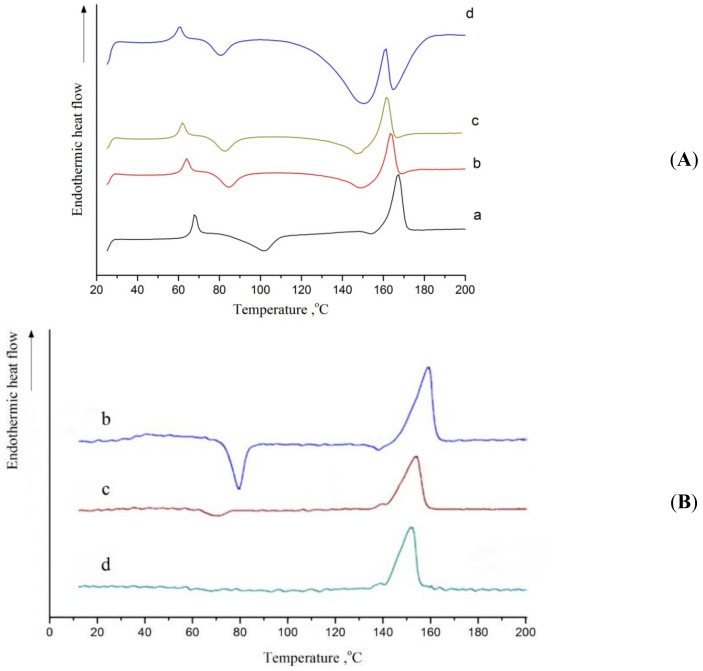
DSC curves of the first heating scan (**A**) and second heating scan (**B**) for pristine PLA fibrous material (a) and PLA fibrous materials with 1% (b), 3% (c) and 5% (d) added OTOA, respectively.

Figure 9A shows the DSC curves of the first heating for the reference fibrous PLA mat and PLA + 1% OTOA, PLA + 3% OTOA and PLA + 5% OTOA samples. In the recorded thermograms, the characteristic low-temperature transition around 65 °C could be attributed to the PLA glass transition (T_g_), while characteristic exothermic and endothermic peaks correspond to the cold crystallization (T_cc_) and the melting (T_m_) of PLA. The respective temperatures (T_g_, T_cc_ and T_m_) are presented in Table 4. The areas of the corresponding peaks were obtained and the enthalpy of PLA cold crystallization (ΔH_cc_) and melting (ΔH_m_) were calculated, together with the degree of crystallinity of the samples (X_c_).

The X_c_ value for the studied PLA fibrous materials was estimated according to the following equation:

X_c_(%) = ((ΔH_m_ − ΔH_cc_)/(ΔH^0^_m_(1 − Φ)) × 100% (6)

where ΔH^0^_m_ is the melting enthalpy of fully crystallized PLA, which is 93 J/g [54] and Φ is the mass fraction of the plasticizer (OTOA). The X_c_ values for the studied PLA samples are shown in Table 4.

The glass transition for the studied PLA samples manifested itself as a combination of a maximum and a characteristic step, corresponding to the second-order thermodynamic transition. The decrease in the peak height for the OTOA-containing PLA samples indicates that its addition to the PLA fiber changed the structure of the amorphous fraction of the polymer formed during the electrospinning process. The exothermic transition in the temperature range of 80–110 °C could be attributed to the cold crystallization of PLA and was observed for all studied PLA samples regardless of the concentration of the added low-molecular-weight OTOA. With an increase in the additive content, this peak shifted to lower temperatures, which is consistent with the plasticizing effect of OTOA [42]. A decrease in the enthalpy of cold crystallization (ΔH_cc_), observed for all OTOA-containing samples, shows that OTOA hinders PLA crystallization, i.e., this compound acts as an amorphising agent. The PLA samples with the OTOA addition crystallized at lower temperatures, as compared to the pristine PLA, leading to a decrease in the temperature interval between the cold crystallization temperature (T_cc_) and the glass transition (T_g_) temperature. According to the previous reports, a decrease in this interval indicates the increase in the plasticizing effect [27,42].

The DSC thermogram for pristine PLA fibers showed an endothermic peak at 167.2 °C, which is characteristic of PLA polymer melting. With an increase in the mass fraction of OTOA in the fiber, a superposition of exo- and endothermic peaks was observed in the 140 –180 °C temperature range. The endothermic peak corresponds to the melting of PLA, while the exothermic peak is due to the complex reaction of the thermal destruction of OTOA by the mechanism of the C-O-O-C bonds breaking and the formation of C-OH [34]. This exothermic reaction is irreversible, which is confirmed by the results of subsequent reheating (Figure 9B) DSC thermograms of the second heating scan for the OTOA-containing PLA samples showed a well-resolved single melting peak in the temperature range of 140–170 °C, without the signs of superposition observed in the DSC thermograms of the first heating. Moreover, as the OTOA content increased, this melting peak showed a decrease in its area (i.e., drop in crystallinity) and a T_m_ shift towards lower temperatures. Both observed effects could be attributed to the plasticizing action of OTOA. In addition, the exothermic peak related to cold crystallization was noticeable in the second heating DSC thermogram only for the PLA + 1% OTOA sample; it was hardly visible for the PLA samples with 3% and 5% OTOA. The absence of the cold crystallization led to the observed decrease in the area of the peak related to PLA melting (Figure 9B).

The data shown in Table 4 gives evidence that the introduction of OTOA additives into PLA fibers led to the plasticizing effect, which was manifested in the decrease in the glass transition temperature from ~67° to ~61°C, a noticeable decrease in the cold crystallization temperature from ~102° to ~81 °C and a slight change in the melting point of the polymer (ΔT_m_ = 4.5 °C). All these phenomena are unambiguously associated with an increase in the segmental mobility of PLA caused by OTOA plasticization. At the same time, a decrease in the enthalpy of cold crystallization was observed upon OTOA addition, together with a decrease in the degree of crystallinity of PLA fibers from 16.70% to 7.4%. Therefore, along with the plasticizing effect of OTOA, it could impede PLA crystallization, as was observed in recent works [16]. This effect could be attributed to the intermolecular interaction between the PLA terminal –OH groups and OTOA molecules observed by FTIR, leading to the steric hindrance for the segmental motion of PLA polymer ends. This immobilization of PLA molecules provides the physical hindrance for PLA crystallization and leads to the decrease in PLA fiber crystallinity upon OTOA addition. The absence of the cold crystallization peak in the second heating endotherm for PLA samples with 3% and 5% OTOA could be accounted for in a similar way.

### 3.4. Mechanical Properties

**Figure 10:**
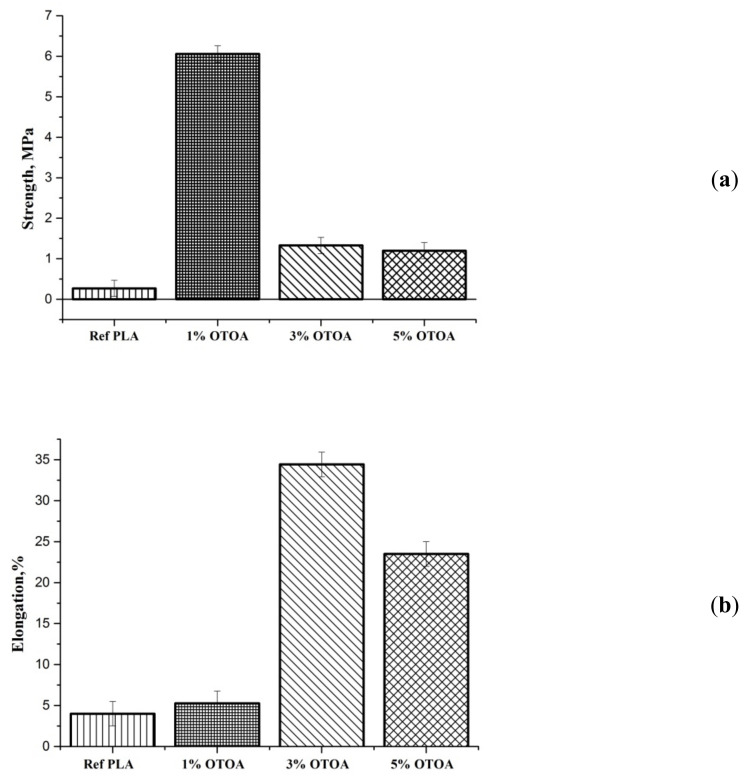
Tensile strength (**a**) and relative elongation at break (**b**) for nonwoven PLA materials: pristine PLA, PLA + 1% OTOA, PLA + 3% OTOA, PLA + 5% OTOA.

Changes in the structure of plasticized PLA fibrous materials have a significant effect on its mechanical characteristics. Figure 10 shows tensile strength (a) and relative elongation at the break (b) for the PLA samples studied. With the introduction of 1% OTOA into PLA, there was a sharp increase in the tensile strength of the material up to 6.2 MPa, as compared to 0.3 MPa value for the pristine PLA. The further increase in the OTOA content up to 3% and 5% led to a decrease in the strength of the material as compared to the PLA + 1% OTOA sample, to 1.5 MPa and 1.2 MPa, respectively. A similar increase in the tensile strength passing through the extremum was previously observed for PLA/cellulose nanowhiskers composites [56], which was attributed to the good dispersion and interaction between the –OH groups of the cellulose nanowhiskers and the C=O groups of the PLA. An enhancement of the tensile properties for the forcespun gelatin/zein and gelatin/PLA/curcumin nanofibers was also recently reported [58,59]. The results showed an improvement in elongation at break and the tensile strength of the forcespun fibers with an increase in the zein concentration in gelatin/zein (1:1–1:4) fibers and an increase in the PLA content in the gelatin/PLA/curcumin composite fibers. The observed effects were attributed to the electrostatic interactions between zein and gelatin and hydrogen bonding between gelatin, PLA and curcumin. It could be concluded that the interaction between PLA and low molecular or polymer additives (hydrogen bonding, polar interactions) leads to improved tensile properties for the nonwoven PLA fiber materials. This could be attributed to the increased level of chain orientation due to interaction of PLA with additives [60].

The effect of the addition of OTOA on the relative elongation was somewhat different (Figure 10b). Thus, the addition of 1% OTOA into the PLA led to a slight increase in this parameter as compared to the pristine PLA. A drastic increase in relative elongation was observed for the PLA + 3% OTOA sample, accompanied by a moderate decrease in this parameter for the PLA sample with 5% OTOA content.

The observed behavior of the mechanical properties directly indicates the plasticizing effect in the PLA fibers upon OTOA introduction. This effect is apparently associated with a change in the structural–dynamic state of the amorphous regions of PLA. In our previous works on the PLA–PHB system, it was shown by the ESR method that the state of the amorphous phase of PLA changes significantly upon the addition of PHB [24]. The addition of small PHB concentrations (up to 30%) to PLA leads to a decrease in the density of amorphous regions [61]. A similar effect should be expected upon the plasticization of PLA with OTOA molecules. The observed decrease in the relative elongation at break for the fibrous PLA mats with the 5% OTOA content could be attributed to the effect of steric hindrance, which the bulky OTOA molecule has on the segmental motion of PLA, provided that this effect exceeds the effect of plasticization. With an increase in OTOA content to 5%, the steric effect begins to prevail over the plasticization effect, increasing the PLA chain rigidity and thus reducing the relative deformation of the PLA fibers.

Summing up the obtained results on the physicochemical properties of electrospun PLA mats modified with OTOA, it could be concluded that it is possible to control the morphology, water sorption capacity, as well as the thermal and mechanical properties of PLA fiber materials via OTOA content. Thus, nonwoven PLA material with 3% OTOA showed a highly porous surface morphology, an increased specific surface area and high-water sorption, together with improved tensile strength and elasticity. PLA fiber material with 1% OTOA showed a greatly increased tensile strength as compared to pristine PLA. FTIR results revealed an interaction between the OTOA and the PLA matrix, wherein OTOA acts as a plasticizer. The plasticizing effect of OTOA, as shown by DSC and mechanical tests, contributed to the thermal and mechanical properties of the PLA mats. At the same time, with a higher content of OTOA (5%), the effects related to the interaction of OTOA with PLA polymer chains and the hindrance of the segmental motion of PLA polymer ends could prevail over the plasticizing effect of OTOA, leading to the deterioration of the morphological and mechanical properties for the PLA fiber material with 5% OTOA content. It could be concluded that the developed PLA material with 3% OTOA content possesses optimal physicochemical properties, namely, improved morphology, mechanical properties and water sorption and could be promising for various biomedical applications, such as drug delivery and tissue engineering. Moreover, since OTOA shows apparent antimicrobial activity [30,31,34], it could be used not only as an efficient plasticizing agent, but as the functional additive as well. Therefore, developed nonwoven PLA-OTOA materials could be used as innovative textile products with antibacterial activity.

## 4. Conclusions

In this article, we have studied the morphological, mechanical, thermal and water sorption properties of electrospun PLA mats after the addition of oleic acid triglyceride ozonide (OTOA). The SEM analysis demonstrated that the fiber morphology changed as a result of the addition of OTOA, which was manifested in the increase in the average fiber diameter, fiber porosity and specific surface area for OTOA-modified PLA mats. The nonwoven PLA material with 3% OTOA content was characterized by a highly porous surface morphology, an increased specific surface area and high water sorption. Contact angle measurements showed that the addition of OTOA leads to the significant hydrophilization of the PLA fiber. FTIR results revealed a weak interaction between the OTOA and the PLA matrix, wherein OTOA acts as a plasticizer and facilitates the segmental mobility of PLA and, thereby, contributes to the thermal and mechanical properties of PLA mats. DSC results showed that OTOA aided the PLA amorphization process, thus reducing the crystallinity of the obtained nonwoven PLA–OTOA materials. Mechanical tests showed that both the tensile strength and the elasticity of electrospun PLA mats were improved by the addition of 3% OTOA. The developed PLA material with an optimal 3% OTOA content could be used in various biomedical applications.

## Figures and Tables

**Table 1:** Average, maximum and minimum fiber diameters for the nonwoven PLA fiber mats with different amounts of added OTOA.

Sample	Average Fiber Diameter, μm	Max. Fiber Diameter, μm	Min. Fiber Diameter, μm
PLA	5.7 ± 2.3	15.0	0.6
PLA + 1% OTOA	9.2 ± 2.5	17.5	0.8
PLA + 3% OTOA	7.8 ± 2.0	14.0	1.6
PLA + 5% OTOA	8.6 ± 3.2	16.0	1.0

**Table 2:** Surface (ρ_s_) and bulk (ρ_v_) density, open volume of the capillaries (V_C_), proportion of the open volume of the capillaries to the total volume of the material (W_C_) and specific surface area (S) for the nonwoven PLA fiber mats with different amounts of added OTOA.

Sample	ρ_s_·10^3^ g/cm^2^	ρ_v_ g/cm^3^	V_C_ cm^3^	W_C_ %	S m^2^/g
PLA	1.5·10^−3^	0.6	7·10^−4^	11.2	0.22
PLA + 1% OTOA	3.9·10^−3^	0.7	7·10^−4^	4.2	2.0
PLA + 3% OTOA	7.0·10^−3^	1.4	9.2·10^−3^	81.7	3.1
PLA + 5% OTOA	3.5·10^−3^	1.0	6.8·10^−3^	57	0.47

**Table 3:** Characteristic bands in the FTIR spectra of PLA and PLA + OTOA fiber materials.

PLA Characteristic Bands, cm^−1^	PLA + OTOA Characteristic Bands, cm^−1^	Characteristic Band Assignment
2995	2995	–CH (asim)
2947	2947	–CH (sim)
2880	2880	CH_3_ stretching
1745	1751	C=O stretching
1455	1455	–CH_3_ bending
	2928	–CH_2_ (asim)
	2856	–CH_2_ (sim)

**Table 4:** Thermal parameters for the studied PLA fibrous materials (first scan).

Sample	T_g_ (°C)	T_cc_ (°C)	ΔH_cc_ (J/g)	T_m_ (°C)	ΔH_m_ (J/g)	X_c_ (%)
PLA	67.3	101.7	21.7	167.2	37.2	16.7
PLA + 1% OTOA	65.2	89.1	14.6	165.5	25.3	11.6
PLA + 3% OTOA	63.4	85.3	15.3	163.3	24.7	10.4
PLA + 5% OTOA	61.3	81.4	15.7	162.7	22.3	7.4
